# First reported cases: renal denervation with secondgeneration multi-electrode catheter via brachial and radial access

**DOI:** 10.5830/CVJA-2015-089

**Published:** 2016

**Authors:** MJ Heradien, PA Brink, MJ Heradien, J Augustyn, A Saaiman

**Affiliations:** Department of Internal Medicine, Stellenbosch University, South Africa; Department of Internal Medicine, Stellenbosch University, South Africa; SA Endovascular, Netcare Kuilsriver Hospital, Cape Town, South Africa; SA Endovascular, Netcare Kuilsriver Hospital, Cape Town, South Africa; SA Endovascular, Netcare Kuilsriver Hospital, Cape Town, South Africa

**Keywords:** hypertension, renal denervation, atrial fibrillation

## Abstract

Renal denervation is a minimally invasive procedure that aims to reduce brain–kidney sympathetic cross-talk. Despite the negative results of the recent SYMPLICITY HTN-3 trial, the procedure is considered safe and has been associated with many beneficial effects, including the reversal of hypertensive heart disease substrate and the prevention of cardiac arrhythmia. The first-generation radiofrequency catheter system featured a monopolar catheter that required sequential singlepoint energy application, followed by rotation, partial withdrawal of the catheter and re-application of energy. The latest generation device features four electrodes configured in a helical arrangement that can simultaneously ablate in four quadrants of the vessel circumference. Renal denervation via brachial or radial arterial access with the second-generation system has not been described before.

## Abstract

Renal denervation (RD) is a minimally invasive procedure that aims to reduce brain–kidney sympathetic cross‐talk. Despite the negative results of the recent SYMPLICITY HTN‐3 trial,^1^ the procedure is considered safe and has been associated with many beneficial effects, including the reversal of hypertensive heart disease substrate and the prevention of cardiac arrhythmia.^2^

The first-generation radiofrequency (RF) catheter system featured a monopolar catheter that required sequential single-point energy application, followed by rotation, partial withdrawal of the catheter and re‐application of energy. The latest generation device features four electrodes configured in a helical arrangement that can simultaneously ablate in four quadrants of the vessel circumference (Fig. 1A). Although the system is designed for femoral access, brachial or radial procedural access has possible advantages, including reduced risk of bleeding and easier access to the renal arteries due to the acute take-off angles of the renal artery from the abdominal aorta.

**Fig. 1A.**
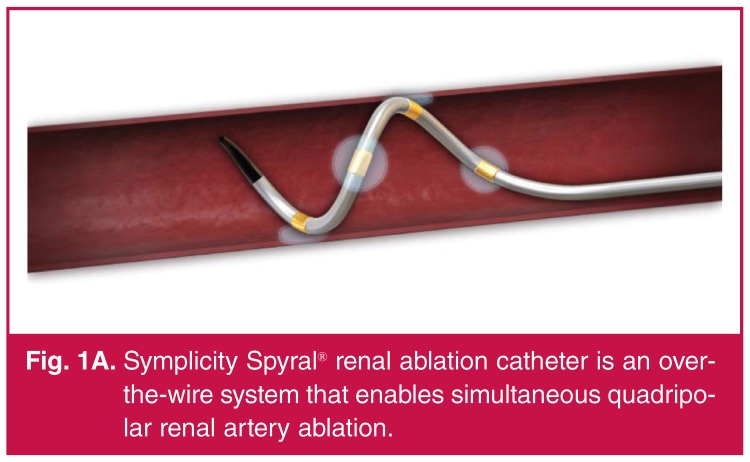
Symplicity Spyral® renal ablation catheter is an overthe‐ wire system that enables simultaneous quadripolar renal artery ablation.

As part of our ongoing trial aiming to determine whether sympathetic modulation with RD can prevent recurrence of atrial fibrillation (‘RDPAF’; clinicaltrials.gov identifier: NCT01990911), we report on two cases of RD with the next generation RD catheter system performed via brachial or radial access. The trial was approved by our local ethics committee, conformed to the Declaration of Helsinki, and the subjects provided written informed consent.

## Case 1: renal denervation via brachial arterial access

Our first case was a 62-year-old female patient (body mass index > 30 kg/m^2^) with a history of uncontrolled hypertension and type 2 diabetes mellitus, and paroxysmal atrial fibrillation managed with rivaroxaban, which was discontinued four days prior to the procedure. Baseline office blood pressure was 150/90 mmHg.

Routine femoral access was achieved. However, catheter access to the right renal artery failed due to the acute anatomical takeoff of the vessel. Therefore, it was decided to attempt access via a brachial approach as an alternative. Percutaneous left brachial arterial access was achieved with a 6-Fr introducer sheath (Terumo), 6-Fr multipurpose guiding catheter (Medtronic) and a 190-cm, 0.014-inch gage BMW^TM^ guide wire (Abbott Vascular). A Symplicity Spyral^TM^ (Medtronic) catheter was then introduced over the guidewire, after removing the straightening tool, resulting in approximately 125 cm of catheter length (Fig. 1B).

**Fig. 1B.**
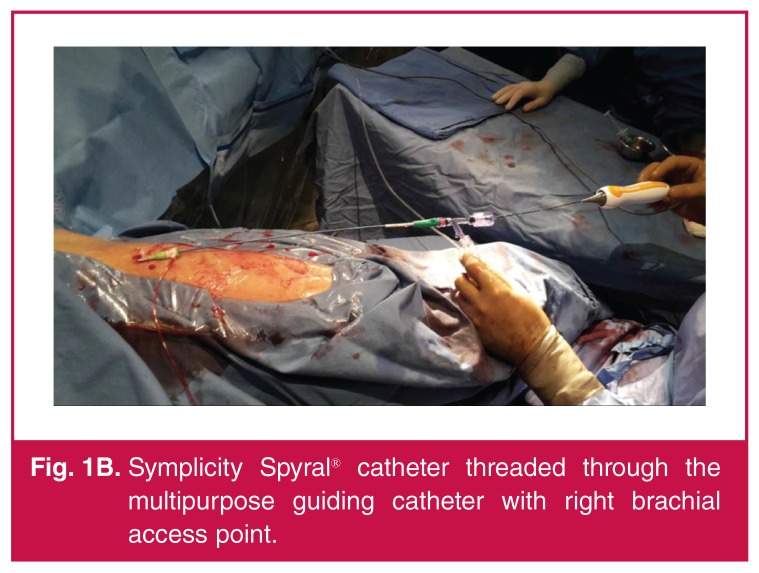
Symplicity Spyral® catheter threaded through the multipurpose guiding catheter with right brachial access point.

**Fig. 2.**
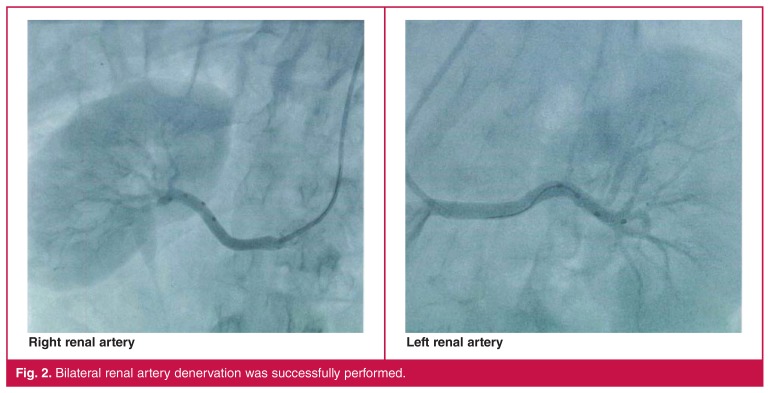
Bilateral renal artery denervation was successfully performed.

The diameter of the main renal artery was approximately 6.5 and 5.5 mm on the left and right side, respectively. Access to both arteries was readily attained, and 17 and 13 lesions were created in the right and left renal arteries, respectively (Fig. 2).

## Case 2: renal denervation via radial arterial access

**Fig. 3.**
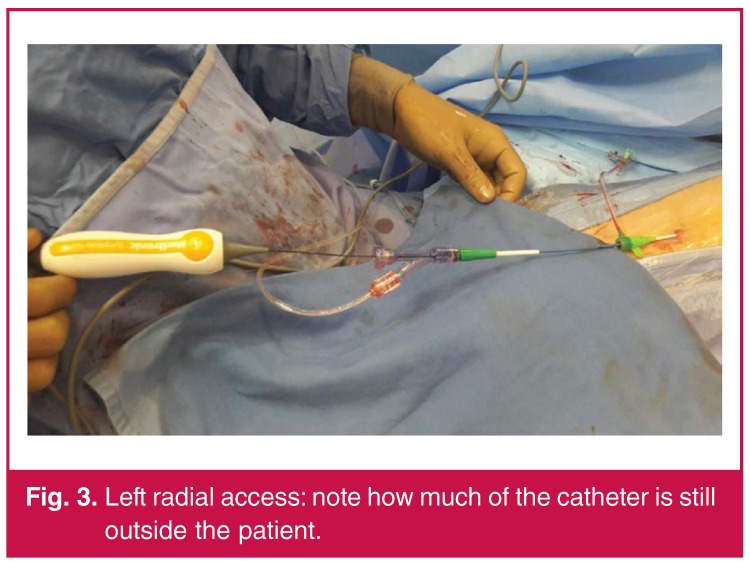
Left radial access: note how much of the catheter is still outside the patient.

The second case was performed on a 63-year-old male with a history of syncope, obstructive sleep apnoea, hypertension and baseline blood pressure of 160/100 mmHg. Percutaneous left radial arterial access was achieved with a 6-Fr introducer sheath (Terumo), 6-Fr multipurpose guiding catheter (Medtronic) and a 190-cm, 0.014-inch gage Thunder^TM^ guide wire (Medtronic). The Spyral catheter was then introduced over the guidewire, after removing the straightening tool, resulting in approximately 125 cm of catheter length (Fig. 3).

The diameter of the main renal artery was approximately 7 and 6 mm on the left and right side, respectively. Adequate catheter access to the renal arteries was attained and 24 and 20 lesions were performed in the right and left renal arteries, respectively.

For both cases electrode temperature, impedance and impedance decreases were in the typical range for all lesions. Typical generator codes indicating sub‐optimal electrode contact were occasionally observed and were addressed during the procedure by successful repeated energy delivery to the specific electrodes. No procedural complications occurred, and the arterial access site was managed post procedurally with routine manual compression.

The patients were discharged the same day as the procedure, and no further complications have been reported to date. Both patients will continue to be monitored according to the clinical protocol (Table 1).

**Table 1:** Comparison: baseline versus follow up after renal denervation (RDN)

	*Before RDN (baseline)*	*Follow up*	*Reduction*
Patient 1: right brachial approach Office BP (mmHg)	05/02/2015: 159/94	12/06/2015: 137/77	22/17 mmHg
ABPM: mean BP (mmHg)	137.1/78.1	130.2/74.9 (4 months after RDN)	6.9/3.2 mmHg
BP meds (3 drugs)	Prexum Plus/Bisocor	Prexum Plus/Bisocor	no
Patient 2: left radial approach Office BP (mmHg)	23/06/2015: 173/94	17/08/2015: 128/73	45/21 mmHg
ABPM: mean BP (mmHg)	155.5/85	133.2/82 (4 months after RDN)	22.3/3 mmHg
BP meds (4 drugs)	Co-Pritor/Bisocor/Spiractin	Co-Pritor/Bisocor/Spiractin	no

## Discussion

To our knowledge, these are the first reported cases of RD through brachial and left radial access, respectively, using the second-generation multi‐electrode RF generation system. Previous case reports have described successful RD via brachial access with the first-generation monopolar system.^3^,^4^ Compared to the traditional femoral approach, trans‐radial or brachial percutaneous procedures for coronary interventions generally have a lower risk of bleeding complications, fewer access site complications and lower hospital costs, and are preferred by patients. Likewise, the unique geometric anatomy of the renal arteries relative to the abdominal aorta may make renal artery access easier from this superior approach.

The Symplycity Spyral catheter measures 117 cm from spiral tip to shaft end, however, we found that removal of the attached ‘straightening sheath’, increased the usable length to 125 cm, which was adequate for these cases. Both cases had encouraging outcomes with no adverse events. During both procedures, we intentionally targeted the distal portion of the main vessel as well as the distal renal artery beyond the main bifurcation because the sympathetic nerves have been shown to be closer to the arterial lumen in these regions.^5^

Note that the patients described here did not have so called ‘treatment-resistant’ hypertension. However, these subjects received RD therapy as part of a separate clinical trial testing the hypothesis that RD therapy may reduce recurrence of atrial fibrillation. Also, note that the system used in these cases was designed specifically for femoral procedural access and this is specified clearly in the product labelling. However, we chose to apply this device in an ‘off-label’ fashion in order to determine the potential to improve the procedural safety and outcome.

## Conclusion

We demonstrated that RD therapy is feasible with the currently approved multi-electrode RF system with either brachial or radial access, although larger prospective studies are required to determine the actual safety and efficacy of this alternative. Such an approach could perhaps reduce the rate of vascular complications associated with femoral access and also allows for same-day discharge. Finally, we suggest that future generations of RD catheter systems be designed with the goal to allow for brachial and radial arterial access.
